# Chitosan and Sodium Alginate Combinations Are Alternative, Efficient, and Safe Natural Adjuvant Systems for Hepatitis B Vaccine in Mouse Model

**DOI:** 10.1155/2016/7659684

**Published:** 2016-07-14

**Authors:** Nourhan H. AbdelAllah, Nourtan F. Abdeltawab, Abeer A. Boseila, Magdy A. Amin

**Affiliations:** ^1^Viral Control Unit, National Organisation for Research and Control of Biologicals (NORCB), Cairo 12654, Egypt; ^2^Department of Microbiology and Immunology, Faculty of Pharmacy, Cairo University, Cairo 11562, Egypt; ^3^National Organization for Drug Control and Research (NODCAR), Cairo 11562, Egypt

## Abstract

Hepatitis B viral (HBV) infections represent major public health problem and are an occupational hazard for healthcare workers. Current alum-adjuvanted HBV vaccine is the most effective measure to prevent HBV infection. However, the vaccine has some limitations including poor response in some vaccinee and being a frost-sensitive suspension. The goal of our study was to use an alternative natural adjuvant system strongly immunogenic allowing for a reduction in dose and cost. We tested HBV surface antigen (HBsAg) adjuvanted with chitosan (Ch) and sodium alginate (S), both natural adjuvants, either alone or combined with alum in mouse model. Mice groups were immunized subcutaneously with HBsAg adjuvanted with Ch or S, or triple adjuvant formula with alum (Al), Ch, and S, or double formulations with AlCh or AlS. These were compared to control groups immunized with current vaccine formula or unadjuvanted HBsAg. We evaluated the rate of seroconversion, serum HBsAg antibody, IL-4, and IFN-*γ* levels. The results showed that the solution formula with Ch or S exhibited comparable immunogenic responses to Al-adjuvanted suspension. The AlChS gave significantly higher immunogenic response compared to controls. Collectively, our results indicated that Ch and S are effective HBV adjuvants offering natural alternatives, potentially reducing dose.

## 1. Introduction

Hepatitis B virus (HBV) is one of the most common viral infectious diseases of the liver and is considered as a major public health problem. HBV is an important occupational hazard for health workers according to the World Health Organization (WHO) [1]. About 240 million people are chronically infected with HBV which is defined as being hepatitis B surface antigen (HBsAg) positive for at least 6 months [2]. HBV infection accounts for 30% of cirrhosis and 53% of primary liver cancer throughout most of the world [2]. Almost 80% of all cases of hepatocellular carcinoma worldwide may be related to HBV which is second to tobacco among known human carcinogens [3].

There is no specific treatment for HBV; however, since 1982 a vaccine against HBV was proven to be safe and effective [1]. The vaccine had been recommended by WHO to be included in the national immunization system. By May 2002, 154 countries had routine infant immunization with hepatitis B vaccine [4]. Although the vaccine is effective in preventing infection, there are limitations which include an estimated five to 15 percent of vaccine nonresponder [5]. In addition, low rates of completion of vaccinations are another problem of current vaccine [6–8]. Many reasons are proposed for poor responsiveness or nonresponsiveness, including concurrent infections or immunocompromised patients or genetic factors as HLA haplotypes or cytokine and chemokine SNPs. In addition to technical errors as intragluteal injection or inappropriate storage conditions (reviewed in [5]), studies have shown potential need for boosters, use of carriers, including preS-epitope, or using more-potent immunogenic adjuvants [5].

Although alum is currently the most commonly used adjuvant with human and veterinary vaccines, it provides some obstacles that need to be resolved. Alum, like any mineral adjuvants, is difficult to manufacture in a physicochemically reproducible way. This failure affects its formulation immunogenicity. In addition, alum cannot be frozen or easily lyophilized as both of these processes cause the collapse of the gel resulting in gross aggregation and precipitation [9]. Alum adjuvant also induces inflammation and local reactions at the site of injection [10], augmenting the production of IgE antibody responses as part of the overall Th2 profile which is not likely to protect against diseases in which Th1 immunity and MHC class I restricted CTL are essential for protection, for example, viral infections, intracellular parasites, or tuberculosis [11].

There is a general tendency on using natural products as potential source of immune modulating compounds [12, 13]. Different compounds derived from natural origin such as plants, microorganism, algae, or insects have been used in the development of new adjuvants for vaccines and drugs for the treatment of other diseases such as allergy and cancer where immune modulating therapies are needed [12–14]. Two natural adjuvant candidates are chitosan and alginate salts. Studies showed significant evidence supporting their use as adjuvants [9, 10, 15]. Chitosan is a natural polysaccharide, which is converted by deacetylation from chitin that is considered the main component of the cell walls of fungi, the exoskeletons of arthropods, the radula of mollusks, and the beaks of cephalopods, including squids and octopuses. Chitosan has been used in several preclinical and clinical studies with good tolerability, excellent immune stimulation, and positive clinical results across a number of infections [11, 12, 14]. Sodium alginate, a naturally occurring polysaccharide, is the sodium salt of alginic acid. It is a gum, extracted from the cell walls of brown algae. Sodium alginate is generally regarded as nontoxic and nonirritant material. It poses many ideal characteristics as it is biodegradable and mucoadhesive polymer that does not produce toxicity in administration which makes it suitable polymer to use in vaccine developments [15]. In the current study, we compared the improvement of the immunogenic response of hepatitis B vaccine using adjuvants system of chitosan and sodium alginate compared to the currently used adjuvant system.

## 2. Materials and Methods

### 2.1. Animals

Male and female Balb/c mice (6–8 weeks of age, 20–30 g) were included in this study and were obtained from VACSERA vivarium, Helwan, Egypt. Animals were housed in accordance with standard laboratory conditions, under controlled environment with temperature 22 ± 3°C, 55 ± 5% humidity, and 12-hour light/dark cycle. Animals were provided with a standard laboratory diet and water ad libitum. The mice were adapted for one week before starting the experiment to their environment. Animals handling was according to guidelines [13, 16]. Permission to conduct the study was obtained from Ethics Committee in Faculty of Pharmacy, Cairo University.

### 2.2. Preparation of Chitosan and Sodium Alginate Solution

Chitosan powder (Sigma-Aldrich, USA) was dissolved in 0.8% (v/v) acetic acid and 0.9% (w/v) saline and then heated at 37°C with constant stirring. Sodium alginate (Sigma-Aldrich, USA) was dissolved in distilled water and heated at 37°C with constant stirring until complete dissolving; then both solutions were sterilized by autoclaving.

### 2.3. Adjuvant Systems and Vaccine Formulations Preparation

The hepatitis B surface (HBsAg) antigen, produced by GSK Biologicals (Rixensart, Belgium), was diluted in phosphate buffer solution (PBS) to a final concentration of 1 *μ*g/mL (Table 1, formulation 2). The different formulations of adjuvanted vaccine combinations are explained in Table 1. In brief, we diluted the HBsAg in PBS with either aluminum hydroxide gel (alum) (Sanofi-Pasteur, France) or chitosan solution or sodium alginate to obtain a final concentration of HBsAg of 0.1 *μ*g/mL. We used alum at concentration of 0.5 mg/mL (Al) or chitosan at concentration of 0.5 mg/mL (Ch) or sodium alginate at concentration of 5 mg/mL (S). Each adjuvant-antigen mixture was shaken for two hours at 25°C to ensure complete absorbtion of the antigen onto the adjuvant system (Table 1, formulations 3–5). The formulations consisting of alum with either chitosan (AlCh) or sodium alginate (AlS) were prepared by addition of alum to the diluted antigen and shaken for one hour; then either chitosan or sodium alginate was added and shaken again for one hour. Finally the combined adjuvanted vaccine formulation of the three adjuvants (AlChS) was prepared by diluting HBsAg then adding alum, chitosan, and sodium alginate consequently with shaking for half an hour after first and second adjuvant addition; then the final mixture was shaken for an additional hour.

### 2.4. Evaluation of the Loading Efficacy of HBsAg in Suspension Vaccine Formulations

The loading efficacy of each vaccine formulation suspension, namely, Al, AlCh, AlS, and AlChS, was calculated indirectly by quantifying the free antigen remaining in the supernatant after the mixture was centrifuged at 10,000 rpm for ten minutes as described previously [17, 18]. The loading efficacy (LE) values were calculated according to the following equations:

LE(%) = (total amount of HBsAg − free HBsAg)/(total amount of HBsAg) *∗* 100.

### 2.5. Experimental Design of Immunization Studies

Balb/c mice were randomly assigned to eight groups (*n* = 6 mice) as shown in Table 1. The groups were control groups (1–3) where group (1) negative control (PBS alone); (2) HBsAg 1 *μ*g/mL in PBS solution (1 *μ*g); and (3) HBsAg 0.1 *μ*g/mL loaded on alum (Al). Groups 4–8 were immunized with the following formulations: group (4) HBsAg 0.1 *μ*g/mL in chitosan solution (Ch); (5) HBsAg 0.1 *μ*g/mL in sodium alginate (S); (6) HBsAg 0.1 *μ*g/mL loaded on alum and chitosan (AlCh); (7) HBsAg 0.1 *μ*g/mL loaded on alum and sodium alginate (AlS); and (8) HBsAg 0.1 *μ*g/mL loaded on alum, chitosan, and sodium alginate (AlChS). Mice groups were inoculated subcutaneously with 1 mL of respective formulations following guidelines for* in vivo* assay [19–22]. Blood samples were collected at four weeks after immunization. Sera were separated by centrifugation at 4000 rpm for ten minutes and stored at −20°C until tested.

### 2.6. *In Vivo* Safety Assay

Each mouse in each group was monitored for 14 days and the toxicity was assessed by survival rate. In addition, local inflammation symptoms as redness, local swelling, and loss of hair at the site of injection were monitored for each mouse.

### 2.7. Measurement of Rate of Seroconversion

The rate of positive seroconversion was measured by using commercial enzyme-linked immunosorbent assay (ELISA) (Diasorin, Italy). The rate of seroconversion was calculated by the following formula:

Rate of seroconversion (%) = (number of mice with specific IgG to HBsAg in their sera that is equal or above 10 mIU/mL)/(total number of mice injected immunized in each group) *∗* 100.

### 2.8. Measurement of Total HBsAg-Specific Antibodies and Antibodies Subclasses

Sera in each mice group were pooled and used for measurement of HBsAg-specific antibodies (IgG) and IgG subclasses. Total IgG was measured using commercial ELISA quantification kit (Diasorin, Italy) as described by the manufacturer and the results were represented as mIU/mL. For measuring HBsAg-specific IgG1, IgG2a, and IgG2b, purified HBsAg (1 *μ*g/mL) was dissolved in 0.05 M carbonate-bicarbonate buffer, pH 9.6, and dispensed into 96-well microtiter plate. Coated plates were incubated at 4°C overnight, washed with PBST (PBS with 0.05% Tween 20) three times, and blocked with 5% bovine serum albumin (BSA) in PBST for two hours at 37°C. After washing the plates with PBST, the sera of each group were added to the wells. Plates were incubated at 37°C for two hours. After washing the plates with PBST, horseradish peroxidase-labelled anti-mouse isotypes (anti-IgG1, anti-IgG2a, and anti-IgG2b) (Komabiotech, Korea) were added and incubated for one hour at 37°C. The plates were washed again with PBST and the bound antibodies were revealed by adding 3,3′,5,5′-Tetramethylbenzidine (TMB) (Sigma, USA). The reaction was stopped with 0.2 M of H_2_SO_4_ and the absorbance was read at 450/630 nm in an automatic ELISA reader (Dynex, USA). ELISA titers were expressed in mIU/mL, where 1 mIU is the mean of absorbance readings for the control group serum plus two times the standard deviation (SD).

### 2.9. Cytokines Measurements

The pooled sera of each group were used for quantification of anti-mouse interferon gamma (IFN-*γ*) and interleukin- (IL-) 4 using commercial ELISA kit (Bosterbio, USA), as recommended by the manufacturer. Cytokines levels were expressed in pg/mL.

### 2.10. Statistical Analysis

Data were analyzed using GraphPad Prism 6.01 (Graph-Pad Software Inc., California, USA). We used one-way analysis of variance (ANOVA) followed by multiple comparisons using Fisher's LSD test for comparison of cytokine and antibody levels means. *P* values less than 0.05 were considered significant.

## 3. Results

### 3.1. Addition of Chitosan and Sodium Alginate Significantly Improved HBsAg Adsorption in Vaccine Formulations

**Figure 1:**
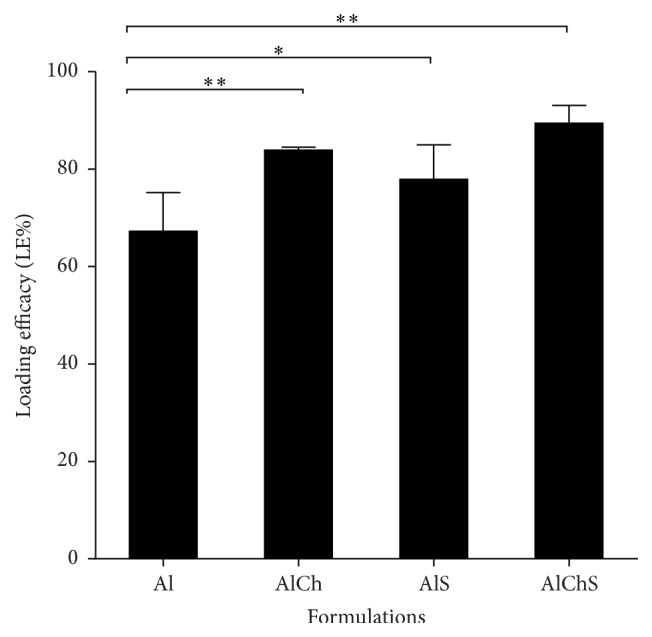
The loading efficacy (LE%) of HBsAg in alum suspension vaccine formulations (Al, AlCh, AlS, and AlChS). Each bar represents mean LE%  ± standard deviation. Natural adjuvant formulations showed significantly higher LE% compared to alum-adjuvanted HBsAg. ^*∗*^ *P* < 0.05 and ^*∗∗*^ *P* < 0.01 compared against alum adjuvant formulation.

The loading efficacy (LE) of HBsAg antigen on alum suspension formulations (AlCh, AlS, and AlChS) was measured to confirm that addition of either chitosan or sodium alginate or both to alum did not decrease the adsorption of antigen to the alum. In contrast the LE% of HBsAg in formulation which contained either chitosan or sodium alginate or both increased significantly from alum alone (*P* < 0.05) (Figure 1). The LE% of Al, AlCh, AlS, and AlChS was 67.72 ± 7.92%, 83.93 ± 0.6%, 77.93 ± 7.04%, and 89.73 ± 3.67% (mean ± SD), respectively (Figure 1). Therefore, new formulations of adjuvant composed of alum with either chitosan or sodium alginate or both showed significantly positive impact compared to alum alone AlCh versus Al (*P* < 0.01), AlS versus Al (*P* < 0.05), and AlChS versus Al (*P* < 0.01) on the adsorption of the antigen (Figure 1).

### 3.2. Natural Adjuvanted Formulations Had No Mortality and Showed No Skin Irritation When Tested in* In Vivo* Model

Mice groups were subcutaneously immunized with different formulations (Table 1) and observed for 14 days. There was no mortality nor weight changes in the immunized animal groups. In addition, we observed that mice immunized with formulations containing natural adjuvants showed no signs for local swelling nor hair loss at the site of injection compared to alum-adjuvanted formulation (data not shown).

### 3.3. Chitosan and Sodium Alginate Showed Comparable Seroconversion to Alum-Adjuvanted Formulation in Mice

**Figure 2:**
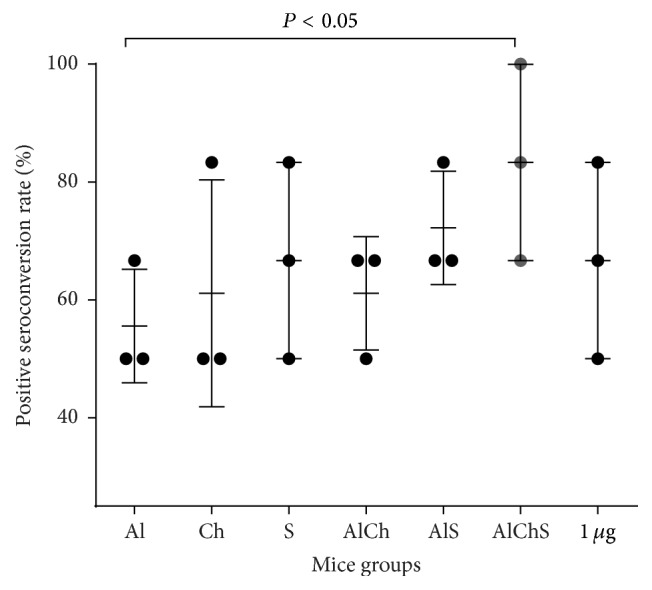
The rate of positive seroconversion expressed in percentage among immunized mice groups. Mice sera were assayed for anti-HBsAg and a cut-off at ≥10 mIU/mL was set as positive. The seroconversion rate was calculated by dividing the number of mice (anti-HBsAg ≥ 10 mIU/mL) on the total number of mice in each group. Data points represent mean ± SD from three independent experiments.

Specific antibodies against HBsAg (anti-HBsAg) of at least 10 milli-international units per milliliter (mlU/mL) are considered a reliable marker of the protective level of immunity [1, 6]. The percentage of mice with sera having anti-HBsAg ≥ 10 mIU/mL divided by the total number of mice vaccinated in each group was evaluated as percent of positive seroconversion rate. All groups showed a percentage of positive seroconversion over 50% (Figure 2). Mice immunized with formulations containing Ch or S or Al as single adjuvants showed no significant difference compared to unadjuvanted control.

### 3.4. Addition of Chitosan and Sodium Alginate to Alum Formula Significantly Increased Seroconversion in Mice Immunized with AlChS Triple Formulation

Mice groups immunized with double adjuvant formulations had no significant difference compared to single adjuvanted formulation or unadjuvanted control (Figure 2). The highest percentage of seroconversion was observed in the triple adjuvant formulation AlChS with seroconversion rate being up to 90% (*P* < 0.05) (Figure 2).

### 3.5. Chitosan and Sodium Alginate Elicited HBsAg-Specific IgG in Immunized Animals

**Figure 3:**
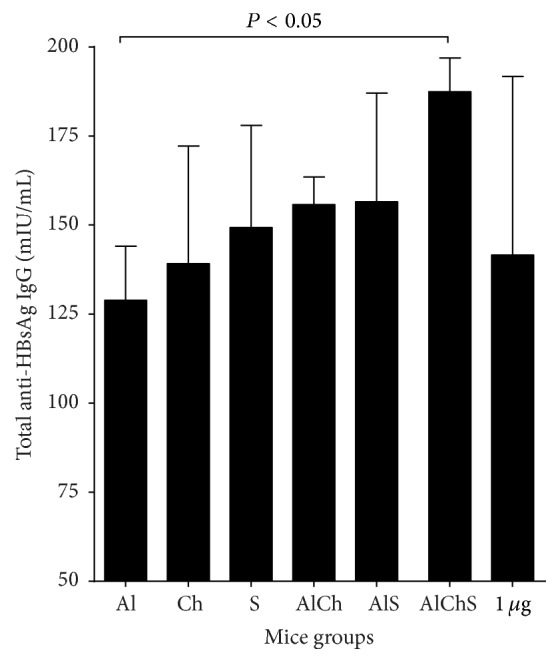
HBsAg-specific IgG titers of mice immunized with different formulations of hepatitis B adjuvanted vaccine. Group of 6 mice was immunized with various HBsAg formulations. Pool of sera for each group collected after 28 days and serum anti-HBs antibody was determined. Each bar corresponds to the group means ± SD from 3 independent experiments.

A pool of the mice sera from each group after 28 days from vaccination were used for determination of total HBsAg-specific IgG levels. In general, the naturally adjuvanted formulations whether single adjuvanted or combined with alum elicited HBsAg-specific IgG comparable to alum-adjuvanted formulation (Figure 3). Moreover, the triple adjuvanted formulation AlChS induced significantly higher HBsAg-specific IgG levels than alum-adjuvanted group (*P* < 0.05) (Figure 3) suggesting some sort of synergism between the three adjuvants.

### 3.6. Formulations Containing Chitosan and Sodium Alginate Elicited Broad Range of Anti-HBsAg IgG Subclasses in Mice

**Figure 4:**
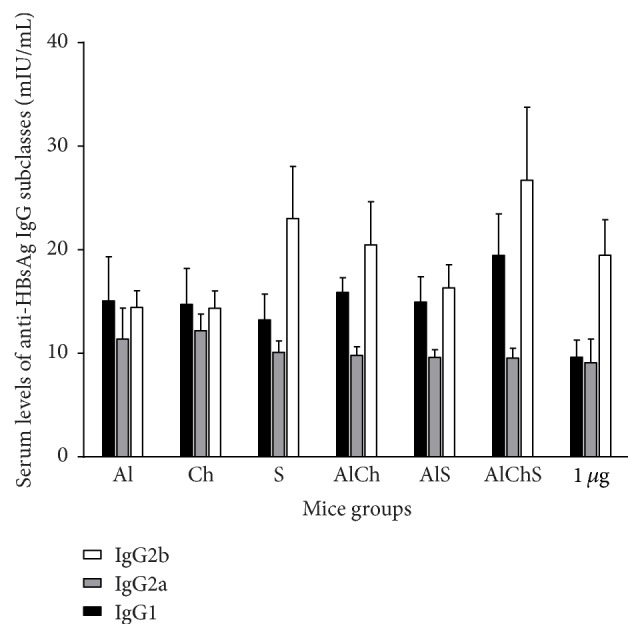
Levels of serum anti-HBsAg IgG subclass elicited in mice immunized with different formulations of the hepatitis B vaccine. Values are expressed as mean of antibody level from 3 independent experiments ± standard variation. *P* < 0.05 (AlChS versus unadjuvanted group) in levels of IgG1; *P* ≤ 0.05 (S versus AlCh group), *P* < 0.01 (AlChS versus AlCh group), and *P* < 0.05 (AlChS versus AlS group) levels of IgG2b.

**Figure 5:**
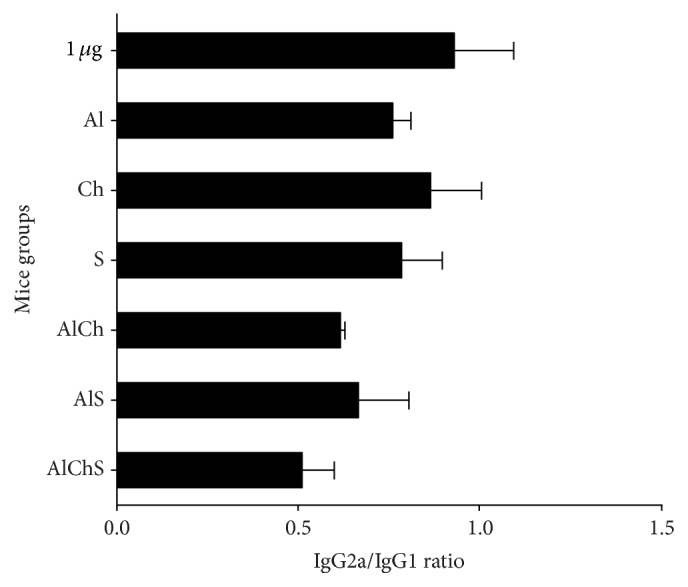
Ratios of IgG2a/IgG1 in mice sera groups immunized with different vaccine adjuvant systems. Ratios depict the percentage of IgG2a divided by the percentage of IgG1. Any ratio >1 is associated with a Th1 response and any ratio <1 is associated with a Th2 response. Each bar represents mean ratio ± standard deviation.

Formulations adjuvanted with Al and Ch elicited IgG1, IgG2a, and IgG2b in mice sera (Figure 4). However, sodium alginate adjuvanted group showed higher serum IgG2b levels compared to the other two groups (Al, Ch) with *P* ≤ 0.05. Similarly, AlS, AlCh, and AlChS adjuvanted formulations elicited a broad range of anti-HBsAg IgG subclasses (Figure 4). All IgG subclasses were elicited in AlChS group, with an increase in IgG1 subclass (Figure 5). In addition, triple formulation showed significant enhancement of IgG2b levels observed in AlChS group especially compared with Al and Ch group (*P* < 0.01) and AlS group (*P* < 0.05) (Figure 4).

### 3.7. Use of Combination of Chitosan and Sodium Alginate with Alum Triple Formulation Elicited Highest IL-4 Response* In Vivo* While Combination of Chitosan or Sodium Alginate with Alum as Double Formulation Elicited Highest IFN-*γ* Response* In Vivo*

**Figure 6:**
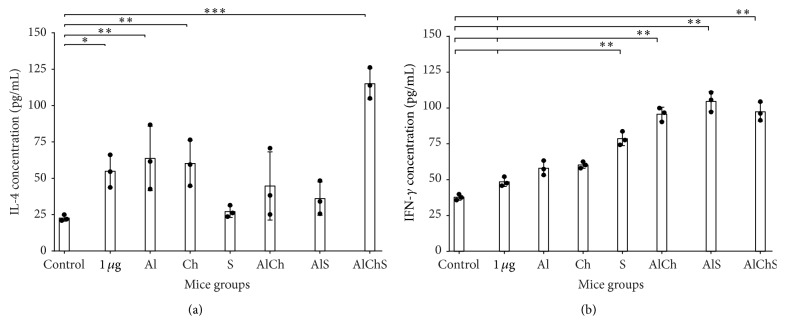
IL-4 and IFN-*γ* levels in mice sera immunized with different formulation of HBsAg. Cytokine values are expressed as means ± SD of three pools. Levels of (a) IL-4 and (b) IFN-*γ* in mice sera immunized with different formulation of HBsAg. ^*∗*^ *P* < 0.05, ^*∗∗*^ *P* < 0.01, and ^*∗∗∗*^ *P* < 0.001 compared to control group.

We evaluated IL-4 and IFN-*γ* to further study immune responses and cytokine production induced by each adjuvant system formulation. Mice groups immunized with single adjuvants of Al and Ch showed comparable levels of IL-4 and significantly higher levels than control (Figure 6(a)). Formulation containing S showed the lowest IL-4 levels that is comparable to control (Figure 6(a)). Meanwhile, double and triple formulations produced significantly higher IL-4 levels than the control and unadjuvanted groups (Figure 6(a)). Mice immunized with triple adjuvant system AlChS elicited the highest levels of IL-4 compared to all groups (*P* < 0.001) (Figure 6(a)).

On the other hand, all adjuvanted groups elicited IFN-*γ* levels higher than unadjuvanted and control groups (Figure 6(b)). The combined adjuvanted groups AlCh, AlS, and AlChS showed higher levels of IFN-*γ* than single adjuvanted groups (*P* < 0.01) (Figure 6(b)). Sodium alginate adjuvanted formulation induced higher IFN-*γ* production compared to alum or chitosan single adjuvanted formulation (*P* < 0.01) (Figure 6(b)). AlS induced the highest IFN-*γ* levels among all adjuvanted formulations (Figure 6(b)).

## 4. Discussion

Hepatitis B vaccine is composed of purified recombinant proteins, which, despite of its better tolerability, is unfortunately less immunogenic when administrated alone. Therefore, there is a constant need to develop new adjuvants to increase the immunogenicity of HBV vaccine so lower doses of HBsAg can be used. In the present study, we compared the safety and immunogenic response of different natural adjuvant systems chitosan, sodium alginate, or both compared to the currently used adjuvant alum. Despite the fact that alum adjuvant has been used in practical vaccination for over 80 years, its drawbacks cannot be ignored. One major drawback is that traditional alum-adsorbed vaccines are frost-sensitive suspensions and thus cannot be lyophilized, hence making transportation and long-term storage a problem. Also alum elicits Th2-driven antibody responses with little Th1-type responses, which restricts the protection against many intracellular pathogens especially virus. In addition, there are safety issues concerning inflammation at the site of injection [23, 24].

Natural products can be a vast source of compounds that modulate immune function [12, 13, 16]. In this study, the potential of some naturally derived compounds was examined to be considered as a natural source of vaccine adjuvants with biological activity equivalent to the current commercially available adjuvants. Both chitosan and sodium alginate are naturally abundant polysaccharides. Chitosan was proven to be safe, nontoxic, nonirritable, nonantigenic, biocompatible, and biodegradable [25, 26]. Similarly, sodium alginate is recognized as a safe food and pharmaceutical ingredient by US Food and Drug Administration (FDA). Sodium alginate is also biodegradable and cheap to produce a stable long shelf life [9, 27]. In our study, chitosan and sodium alginate adjuvanted HBV vaccine showed to be safe whether used alone as single adjuvant or in combination with alum. However, our* in vivo* safety assessment would need to be conducted for formulations containing alum over longer periods of time to observe any possible long-term neurological or systemic adverse effects known to be associated with alum.

We first examined the replacement of alum with either chitosan or sodium alginate in the single solution adjuvanted formulation. Both chitosan and sodium alginate adjuvanted vaccine gave comparable immunogenic response to alum and to each other. This was evident by the rate of the positive seroconversion in immunized mice that provided above 50% of anti-HBsAg that is at least 10 mIU/mL (Figure 2). In addition, Ch and S elicited similar levels of total anti-HBsAg IgG (Figure 3); moreover Ch elicited mixed Th1-Th2 IgG subclasses similar to Al (Figure 4). Meanwhile, S elicited more IgG2b, a more Th-1-like response (Figure 4); also S elicited the least IL-4 levels and one of the highest IFN*γ* in mice sera (Figure 6).

It has been shown that Th1 and Th2 responses could be characterized by their cytokine production [28]. The Th1 immune response is indicated by the production of IFN-*γ* and production of IgG2a and IgG2b in mice while the Th2 immune response is indicated by the production of IL-4 and enhanced production of IgG1 (Figures 4 and 6) [19, 29]. The ratio of IgG2a/IgG1 of all formulations indicated a more IgG1, Th2 response (Figure 5). However, sodium alginate group induced high level of IgG2b versus alum and chitosan group which indicate a tendency toward Th1-like response. Overall, this indicates that there is a variety of IgG subclasses elicited by natural alternative adjuvant systems, which offers more protection against hepatitis B. Another marker of Th1/Th2 response is IgG2a/IgG1 ratio. It provides an indication of the Th1/Th2 bias of the ongoing immune response [28]. Mice immunized with chitosan adjuvanted formulation exhibited the highest ratio among all groups which gave a good indication of a balanced Th1/Th2 immune response as indicated by the increase in IgG1, IgG2a, and IgG2b antibody isotypes (Figures 4 and 5). Overall these findings are not far from what was reported in using chitosan with different types of antigen and different route of administration [11, 20, 21]. For example, adjuvanted chitosan solution with OVA antigen when injected parentally in mice produced similar levels of IgG1, IgG2a, and IgG2b [20]. Meanwhile, chitosan solution with* Helicobacter pylori* antigen administrated orally gave IgG2a/IgG1 of 1.06 ± 0.4 which is similar to our result 0.9 (Figure 5) [21].

Our single adjuvant systems results are also in agreement with previous studies where sodium alginate elicited response shifted toward Th1-like response in sodium alginate adjuvanted vaccine of Bacillus Calmett-Guerin (BCG) administrated subcutaneously in mice [15]. We also found a significant increase in the production of IFN-*γ* but a decrease in the production of IL-4 in sodium alginate adjuvanted vaccine compared to alum and chitosan groups which had approximate levels in both IL-4 and IFN-*γ* (Figure 6). These results were consistent with those found in other studies using alginate encapsulated influenza virus preserving its immunogenicity and stimulating potent CD8+ T cell responses by secretion of antiviral cytokines, such as IFN-*γ* [22]. These findings supported the use of either chitosan or sodium alginate alone as a replacement natural safe adjuvant instead of alum for hepatitis B vaccine.

Next, we used chitosan and sodium alginate in combination with alum as either double or triple formulation to enhance the potency of the vaccine by protecting the antigen from degradation* in vivo*, trying to decrease the amount of HBsAg and alum used, thus a more cost-effective vaccine [30, 31]. We found that double and triple formulation offered best immunogenic responses. This was evident as triple formulation had the highest rate of seroconversion (Figure 2) and elicited highest total IgG levels with a mixed IgG1, 2a, and 2b giving a more comprehensive protection (Figures 3 and 4). Finally, triple formulation showed significantly highest level of Th2 cytokine IL-4 and was second to AlS in IFN*γ* levels (Figure 6). Our data supports the use of combined adjuvants to decrease the amount of alum and HBsAg used. This is in line with many of the successful examples as AS04 containing aluminum and the bacterial lipid, monophosphoryl lipid A [32]. This adjuvant system is already licensed in Europe and used in many vaccines like HBV vaccine (Fendrix®) and Human Papilloma Virus (HPV) vaccine (Cervarix®).

Another important parameter was to evaluate the adsorption efficacy of the alum-adjuvanted vaccine (Figure 1). Adsorption of antigen to aluminum-containing adjuvants prior to administration is essential for the enhancement of immunogenicity and essential in avoiding fast degradation of the antigen after administration [33, 34]. In addition, the degree of adsorption became one of the parameters for evaluation of the efficacy of the final vaccine product [33, 34]. Addition of either chitosan or sodium alginate to alum provided efficient loading of the antigen on the adjuvant system (Figure 1). These results are in line with that reported when different formulation of hepatitis B antigen was efficiently associated with alginate coated chitosan nanoparticles with loading efficacy equal to 77.1 ± 3.0% [28]. However in our studies the presence of alum increased the loading efficiently when combined with either chitosan or sodium alginate adjuvant and reached its maximum when the three adjuvants combined together in the AlChS formulation (Figure 1). As expected between all adjuvant formulations in this study, the three-adjuvant combined group (AlChS) stands out as the most immunogenic formulation (Figures 3, 4, and 6).

While addition of chitosan or sodium alginate to alum in a combined adjuvant system gave comparable results to each other on the level of HBsAg-specific IgG antibodies, IgG isotypes, and cytokine production, their immunogenic effect did not increase significantly compared to the single adjuvanted vaccine group. Similar observation was found when both antigen and another adjuvant (CpG ODN 1826) were adsorbed to chitosan nanoparticles as the formulation did not give additional important benefit compared to other formulations containing unadjuvanted antigen and single adjuvant (CpG ODN 1826) formulation [17].

Additionally, in the current study, a group of mice has been immunization subcutaneously with 1 *μ*g HBsAg unadjuvanted, which on its own induced low but detectable anti-HBs. When we reduced unadjuvanted HBsAg doses ten times (0.1 *μ*g) or to 0.5 *μ*g/mL, we found that there were no responses (data not shown). All adjuvanted vaccine formulation induced equivalent peak titers of anti-HBsAg as unadjuvanted vaccine (1 *μ*g), about 10-fold higher with ten times less antigen (0.1 *μ*g), except for AlChS group which induced even more anti-HBs titer than 1 *μ*g HBsAg (Figure 3). Interestingly, regarding IFN-*γ* levels, all adjuvanted vaccine formulations were significantly higher than 1 *μ*g unadjuvanted vaccine which seemed particularly strong in the presence of sodium alginate whether alone or in combination (Figure 3), while the IL-4 levels of 1 *μ*g unadjuvanted group were comparable with the less antigen adjuvanted group with the exception of the group adjuvanted with sodium alginate which produce lower IL-4 (Figure 6). On the contrary AlChS group produce significant higher IL-4 level about 2-fold than produced by the 1 *μ*g unadjuvanted vaccine (Figure 6). Similar observation was obtained with the same mice strain using the same antigen (HBsAg) with alum alone or combined with different adjuvant (CPG ODN) [35]. This result indicates that both chitosan and sodium alginate could induce 10-fold immunogenic response compared to the same dose of unadjuvanted vaccine whether used alone or in combination with alum.

## 5. Conclusions

Our study revealed that natural products can be used as an alternative safe, highly immunogenic adjuvant to HBV vaccine. It is advantageous to use natural alternatives with good biocompatibility and immunological activity on the specific cellular and humoral immune responses to HBsAg in mice. Collectively our results suggested that the use of natural adjuvants, chitosan or sodium alginate, as single adjuvant or in combination with alum can help in production of lower dose of HBV vaccine that can potentially be less expensive. The combined formulation of triple adjuvant where the antigen is enclosed in the adjuvant system with strong adsorption showed strong impact on the immune response. These responses were expressed with the highest level in the cytokine levels, rate of seroconversion, anti-HBsAg, and their isotypes with balanced Th2 and Th1. Our study provides a great opportunity for the improvement of the currently licensed HBV vaccines. However, more studies are needed to assess the long-term safety and application of our findings in clinical settings.

## Figures and Tables

**Table 1:** Composition of adjuvant systems formulations used in the study.

	1 (Negative control)	2 (Unadjuvanted)	3 (Al)	4 (Ch)	5 (S)	6 (AlCh)	7 (AlS)	8 (AlChS)
PBS	x							
HBsAg (1 *μ*g/mL)		x						
HBsAg (0.1 *μ*g/mL)			x	x	x	x	x	x
Alum (0.5 mg/mL)			x			x	x	x
Chitosan (0.5 mg/mL)				x		x		x
Sodium alginate (5 mg/mL)					x		x	x
